# Investigation of total cerebellar and flocculonodular lobe volume in Parkinson’s disease and healthy individuals: a brain segmentation study

**DOI:** 10.1007/s10072-024-07509-5

**Published:** 2024-04-16

**Authors:** Merve Nur Ozgen, Necati Emre Sahin, Nurcan Ertan, Bunyamin Sahin

**Affiliations:** 1 Department of Anatomy, Faculty of Medicine, Tokat Gaziosmanpaşa University, Tokat, Türkiye; 2 Department of Anatomy, Faculty of Medicine, Karabük University, Karabük, Türkiye; 3 Radiology Clinic, Ankara Etlik City Hospital, Ankara, Türkiye; 4 Department of Anatomy, Faculty of Medicine, Ondokuz Mayıs University, Samsun, Türkiye

**Keywords:** Cerebellum, Volume, Magnetic resonance, Parkinson’s disease, VolBrain

## Abstract

**Background:**

Parkinson’s disease (PD) is a neurodegenerative disorder with an unexplored link to the cerebellum. In the pathophysiology of balance disorders in PD, the role of the flocculonodular lobe (FL) is linked to the impairment of the dopaminergic system. Dopamine deficiency can also lead to changes in cerebellum functions, disrupting balance control. This study compares cerebellar and FL volumes between healthy controls (HC) and PD patients, analyzing their correlation with clinical outcomes.

**Methods:**

We used magnetic resonance images of 23 PD patients (14 male, 9 female) and 24 HC (9 male, 15 female). Intracranial (ICV), total cerebellar, FL, and cerebellar gray matter volumes were measured using VolBrain. Clinical outcomes in PD patients were assessed using the Unified Parkinson’s Disease Rating Scale (UPDRS-III) to evaluate motor function, with scores correlated to volumetric data.

**Results:**

The cerebellar and gray matter volumes in HC were 115.53 ± 10.44 cm^3^ and 84.83 ± 7.76 cm^3^, respectively, compared to 126.83 ± 13.47 cm^3^ and 92.37 ± 9.45 cm^3^ in PD patients, indicating significantly larger volumes in PD patients (*p* < 0.05). The flocculonodular lobe gray matter volume was 1.14 ± 0.19 cm^3^ in PD patients and 1.02 ± 0.13 cm^3^ in HC, but there was a significant increase in gray matter volume in PD patients between the groups (*p* < 0.05). In PD patients, significant negative correlations were observed between FL volume and the UPDRS-III scores (*r* =  − 0.467, *p* = 0.033) and between UPDRS-III scores and both total (*r* =  − 0.453, *p* = 0.039) and normalized (*r* =  − 0.468, *p* = 0.032) gray matter volumes of the FL.

**Conclusion:**

Although total gray matter volumes were larger in PD patients, the volumes of FL did not differ between groups. In Parkinson’s disease, increased cerebellar volume may regulate fine motor movements rather than balance.

## Introduction

Parkinson’s disease (PD) is the second most common neurodegenerative disorder after Alzheimer’s disease [1]. PD is a chronic and progressive illness that manifests with both motor and non-motor symptoms. The motor symptoms include bradykinesia, rigidity, tremor, and postural instability; non-motor symptoms include mood disorders, cognitive impairment, sleep disturbances, gastrointestinal dysfunction, and pain [2]. Generally, the underlying mechanism of PD is the neurodegeneration of the basal ganglia. Specifically, it is associated with the loss of dopaminergic neurons in the substantia nigra’s pars compacta. However, this is not sufficient to explain the non-motor symptoms of the disease [2, 3].

In recent years, it has been suggested that PD is not only limited to a deficiency of brain dopamine but involves also other structures within the brain and that the disease represents a complex neurodegenerative process [4, 5]. Recent studies have shown direct synaptic connections between the cerebellum and the basal ganglia and indirect connections at the cortical level. The cerebellum, along with the basal ganglia, is involved in regulating motor coordination and non-motor functions [3, 6]. The flocculonodular lobe (FL) is the lowermost part of the cerebellum, playing a critical role in balance and ocular movement functions. The role of the FL in the pathophysiology of balance disorders in PD is intricately associated with the impairment of the dopaminergic system. The loss of dopaminergic neurons within the substantia nigra in PD can lead to a decrease in cerebral blood flow and oxygenation, as well as alterations in neuronal activity. This dopamine deficiency may, in turn, induce changes in the functions of the cerebellum, impairing balance control [7, 8]. Furthermore, the pathophysiology of the FL has been a critical area of investigation in a variety of neurological diseases [9].

Although PD is clinically diagnosed, magnetic resonance imaging (MRI) can reveal signal changes or atrophy in certain brain regions. Furthermore, MRI facilitates the in vivo diagnosis of PD patients, allows for accurate measurements from images, and assists in understanding morphological changes [10–13]. Parkinson’s disease–related pathological changes are observed in the cerebellum [14]. This situation suggests that the balance disorders seen in  PD patients may be related to the basal ganglia and the cerebellum. In recent years, an excess of volumetric studies have been conducted using various methods and measurement techniques on magnetic resonance (MR) images [12, 15]. However, the results of studies on the cerebellum could be more consistent [12, 15–17]. This study aims to compare the total cerebellar volume and the volume of the FL between healthy controls (HC) and PD patients using MR images. In addition, volume values normalized by the total cerebellum volume and gray matter volume ratio to the  intracranial volume (ICV) were also compared between HC and PD patient groups, to analyze the relationship between the volume data of PD patients and clinical data.

## Methods

### Study population

This study was initiated with approval number 10940098–51 from the non-interventional clinical research ethics committee of Istanbul Medipol University. The study was conducted through a retrospective analysis of patient data from the hospital archive system. Parkinson’s patients included in this study were selected according to the “United Kingdom Parkinson’s Disease Society Brain Bank” criteria [18]. Diagnoses of PD patients were confirmed by a neurologist based on clinical examination and anamnesis. Furthermore, participants were assessed with the clinical dementia rating (CDR) scale [19] to ensure the absence of cognitive impairment. Clinical data of PD patients were recorded as disease duration years, Unified Parkinson’s Disease Rating Scale (UPDRS-III), and Hoehn and Yahr stage (H&Y). A healthy control group was randomly selected to be compatible in age with the PD patients, but anamnestic familial criteria not applied. Care was taken to ensure that individuals in the HC did not have a neurological or psychiatric disease history. This study included 24 HC (9 male, 15 female) and 23 PD patients (14 male, 9 female) aged between 49 and 79.

### Data collecting

Brain MRI images used in the study were collected from the imaging database of the Radiology Department of Istanbul Medipol University. The images obtained with a 3T MRI machine (Philips Achieva TX, the Netherlands) are in DICOM format. T1-weighted structural brain scans were taken. Features of the received image were as follows: matrix = 228 × 227 pixels, field of view = 250 cm × 250 cm, voxels: = 1 mm × 1 mm × 1 mm, TR = 7.9 ms, TE = 3.7 ms, TFE = 97, and flip angle = 8°. Total imaging duration time was 04:33 min. These scans were obtained as a dataset consisting of slices 1 mm thick and comprising 190 slices in total.   Collected images have been anonymized by privacy and ethical rules, keeping participants’ personal information confidential. Brain MR images in DICOM format were converted into compressed NIfTI (nii.gz) format (Neuroimaging Informatics Technology Initiative) using the dcm2nii software (v.1.2, July 2022) from the MRIcroGL package.

### Volumetric analysis

**Fig. 1:**
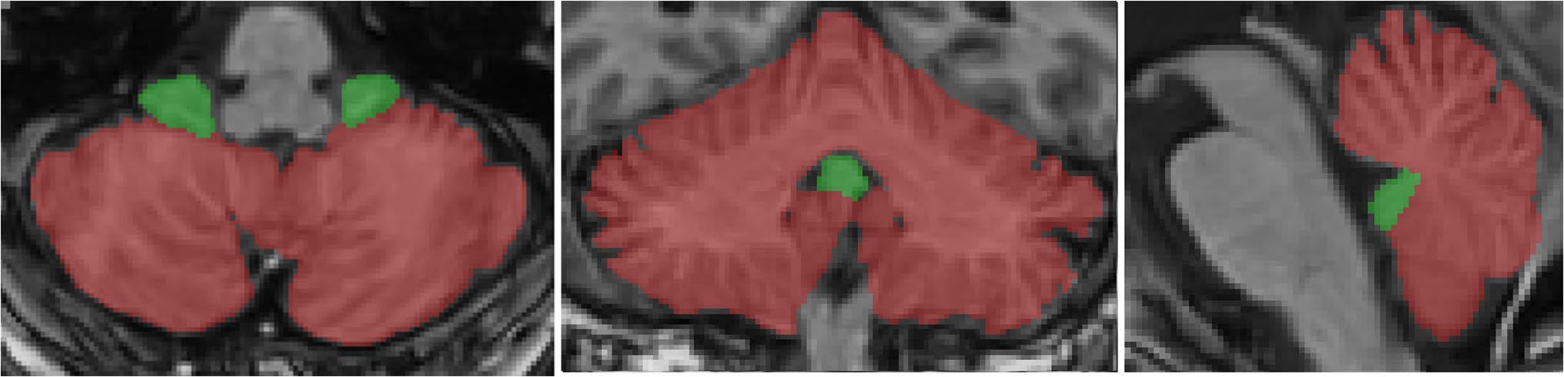
Cerebellar segmentation. Green color indicates the flocculonodular lobe, while red color represents other segments of the cerebellum

**Fig. 2:**
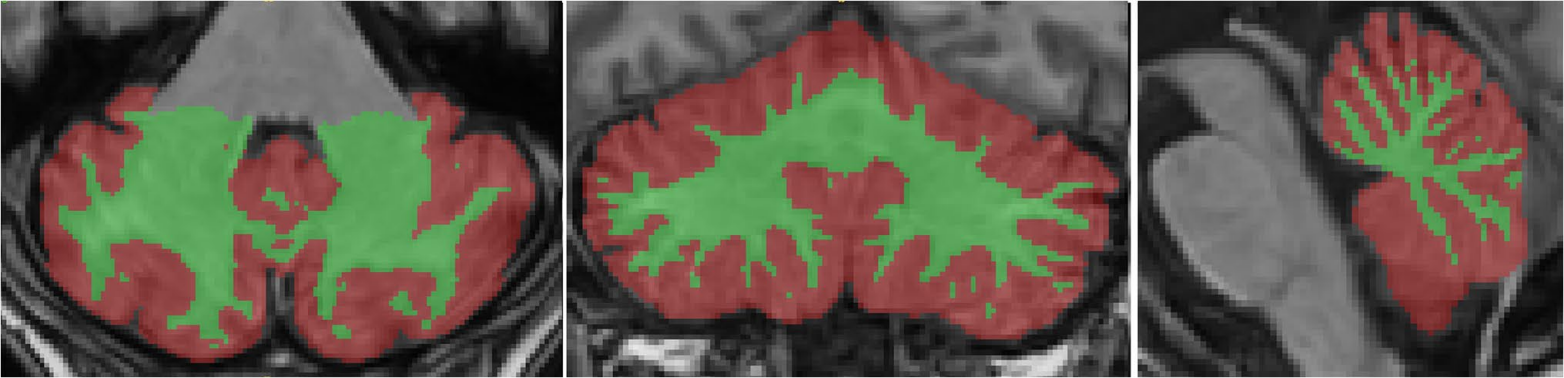
Cerebellar gray matter/white matter tissue classification. Green color indicates white matter, while red color represents gray matter of the cerebellum

The anonymized NIfTI format brain MRI images were processed using VolBrain, a web-based software developed for the automatic processing of brain images and the volumetric analysis of brain structures. The volumetric data related to the cerebellum was obtained using a module called CERES, which is part of VolBrain. CERES is a package that produces reliable results for cerebellar lobule segmentation and volume calculation [20] (Figs. 1 and 2).

Using the CERES module, the following data were obtained: ICV, total cerebellar volume, total volume of the FL, total cerebellar gray matter volume, and total gray matter volume of the FL. The total and gray matter volumes of the cerebellum were normalized by dividing them by the  ICV  and multiplying by 100, yielding their ICV-relative percentages. Similarly, normalization of the total FL volume and total FL gray matter volume involved dividing them by the total volume of the cerebellum and multiplying by 100, resulting in the normalized total FL volume and total FL gray matter volume.

### Statistical analysis

For the statistical analysis of the volumetric data obtained from the research and the demographic data of the individuals, SPSS software (Statistical Package for the Social Sciences, version 25) was utilized. Descriptive statistical methods, such as count (*n*), mean, and standard deviation (SD), were employed to analyze the data. The Spearman correlation test was used to evaluate the relationship between clinical and volumetric data without correction for multiple comparisons. The ages of the individuals and volumetric data were evaluated using the Mann–Whitney *U* test for comparisons between the HC and PD patient groups, and results were considered statistically significant at a level of *p* < 0.05.

## Results

### Population

The study included MRI images from 24 HC (9 male, 15 female) and 23 PD patients (14 male, 9 female). The average age was calculated to be 59.62 ± 7.34 for the HC group and 58.91 ± 6.53 for the PD patient group, with no significant difference found between the age averages of the groups (*p* = 0.890). The mean values of the clinical data of PD patients were as follows: disease duration 4.61 ± 3.80 years, UPDRS-III 15.62 ± 6.02, and H&R 1.52 ± 0.51.

### Comparison of volumes

In this study, the volumes of the cerebellum and FL in the PD patient group were compared with those in the HC group.

In comparing HC (*n* = 24) with PD patients (*n* = 23), the total cerebellar volume showed a significant difference, with HCs having a mean volume of 115.53 cm^3^ and PD patients presenting a larger mean volume of 126.83 cm^3^, which was statistically significant (*p* = 0.006). When considering the normalized total cerebellar volume as a percentage of ICV, HCs had a mean of 8.67% compared to PD patients with a mean of 9.23%, with the increase statistically significant (*p* = 0.008). This analysis suggests that PD patients exhibit a significantly larger total cerebellar volume and a higher percentage of cerebellar volume relative to ICV than healthy controls, highlighting potential cerebellar involvement in PD. The details are given in Table 1.

**Table 1:** Comparison of volume and normalized volume data between groups

	HC (*n* = 24) Mean ± SD	PD patients (*n* = 23) Mean ± SD	*p*
ICV (cm^3^)	1338.09 ± 140.47	1379.01 ± 152.38	0.338
Total cerebellar volume (cm^3^)	115.53 ± 10.44	126.83 ± 13.47	0.006*
Total FL volume (cm^3^)	1.29 ± 0.71	1.29 ± 0.20	0.120
Normalized total cerebellar volume (%)	8.67 ± 0.67	9.23 ± 0.75	0.008*
Normalized total FL volume (%)	1.11 ± 0.60	1.01 ± 0.10	0.670

*ICV* intracranial volume, *FL* flocculonodular lobe, *p < 0.05

In the comparison between HC and PD patients, it was found that PD patients had a significantly higher total cerebellar gray matter volume, with means of 84.83 cm^3^ for HCs and 92.37 cm^3^ for PD patients (*p* = 0.011). Similarly, the total FL gray matter volume was greater in PD patients, with HCs showing a mean of 1.02 cm^3^ and PD patients a mean of 1.14 cm^3^ (*p* = 0.045). When looking at normalized volumes, the total cerebellar gray matter volume as a percentage was also significantly higher in PD patients at 6.73% compared to 6.36% in HCs (*p* = 0.020). These findings suggest that PD is associated with increased cerebellar and FL gray matter volumes, indicating potential cerebellar involvement. The details are given in Table 2.

**Table 2:** Comparison of gray matter volume data between groups

	HC (*n* = 24) Mean ± SD	PD patients (*n* = 23) Mean ± SD	*p*
Total cerebellar gray matter volume (cm^3^)	84.83 ± 7.76	92.37 ± 9.45	0.011*
Total FL gray matter volume (cm^3^)	1.02 ± 0.13	1.14 ± 0.19	0.045*
Normalized total cerebellar gray matter volume (%)	6.36 ± 0.50	6.73 ± 0.54	0.020*
Normalized total FL gray matter volume (%)	1.20 ± 0.10	1.23 ± 0.14	0.431

*FL* flocculonodular lobe, *p < 0.05

### Correlations between volume and clinical data

When the PD patient group analyzed the relationship between volume data and clinical data, a significant negative correlation was found between FL volume and UPDRS-III (*r* =  − 0.467, *p* = 0.033). The details are given in Table 3.

**Table 3:** Correlations of volumes and clinical data in the  PD patients group (*n* = 23)

		UPDRS-III	H&Y	Disease duration (years)
ICV (cm^3^)	*r*	− 0.393	− 0.001	− 0.129
	*p*	0.078	0.998	0.556
Total cerebellar volume (cm^3^)	*r*	− 0.176	0.108	− 0.026
	*p*	0.446	0.643	0.906
Total FL volume (cm^3^)	*r*	− 0.467*	− 0.107	0.058
	*p*	0.033*	0.645	0.791
Normalized total cerebellar volume (%)	*r*	0.072	0.005	0.219
	*p*	0.758	0.983	0.316
Normalized total FL volume (%)	*r*	− 0.428	− 0.242	0.122
	*p*	0.053	0.290	0.579

*ICV* intracranial volume, *FL* flocculonodular lobe, *UPDRS* Unified Parkinson’s Disease Rating Scale, *H&Y* Hoehn and Yahr stage, *p < 0.05

When the relationship between gray matter volume data and clinical data in the PD patient group was examined, significant negative correlations were found between UPDRS-III and both the total gray matter volume of the FL (*r* =  − 0.453, *p* = 0.039) and the normalized total gray matter volume of the FL were found (*r* =  − 0.468, *p* = 0.032). The details are given in Table 4.

**Table 4:** Correlations of gray matter volumes and clinical data in the  PD patients group (*n* = 23)

		UPDRS-III	H&Y	Disease duration (years)
Total cerebellar gray matter volume (cm^3^)	*r*	− 0.267	0.008	− 0.024
	*p*	0.242	0.971	0.912
Total FL gray matter volume (cm^3^)	*r*	− 0.453*	− 0.198	0.061
	*p*	0.039*	0.389	0.781
Normalized total cerebellar gray matter volume (%)	*r*	− 0.133	− 0.071	0.296
	*p*	0.564	0.759	0.170
Normalized total FL gray matter volume (%)	*r*	− 0.468*	− 0.301	0.184
	*p*	0.032*	0.185	0.400

*FL* flocculonodular lobe, *UPDRS* Unified Parkinson’s Disease Rating Scale, *H&Y* Hoehn and Yahr stage, *p < 0.05

## Discussion

In this study, we compared normalized values of the total volume of the cerebellum, the volume of the cerebellar gray matter, and the volume of the FL, each scaled to ICV, between 24 HC and 23 PD patients. The relationship between the results obtained and clinical data was analyzed.

While most studies on postural and balance disorders in PD have focused on the frontal lobe and basal ganglia, cerebellar activity has often been unnoticed [21]. In recent years, the number of studies investigating the involvement of the cerebellum in PD has been increasing. By examining the localization of motor-related activity in the cerebellum using functional magnetic resonance imaging (fMRI), information can be obtained about whether there is hyperactivation or hypoactivation in the cerebellum. Such findings provide new evidence supporting the hypothesis that hyperactivation in the cerebellum may serve as a compensatory mechanism for defective basal ganglia [22]. However, hyperactivation in the cerebellum in PD remains unclear. Although the cerebellum could potentially be related to the symptoms of PD, data on the interaction between the cerebellum and PD in the literature are limited. Conflicting results have been obtained in publications assessing cerebellar volumes through volumetric MRI measurements [15, 16, 23]. This study addressed these controversial findings by comparing HC with PD patients regarding volumetric MRI results and examining the relationship with the FL.

According to our findings, the total cerebellar volume and gray matter volume in PD patients were larger than those in healthy individuals. While unnormalized data showed no difference in ICV and total volume of the FL between groups, the total cerebellar volume, total cerebellar gray matter volume, and total gray matter volume of the FL were larger in PD patients compared to HC. When normalized for ICV, the total volume of the cerebellum and the total gray matter volume of the cerebellum were larger in PD patients compared to healthy individuals. The total volume of the FL and its total gray matter volume, when normalized to the total volume of the cerebellum, did not differ between groups. In the PD patient group, a significant negative correlation was found between UPDRS-III and FL volume, total gray matter volume of FL, and normalized total gray matter volume of FL.

Bharti et al. [24] reported that in a voxel-based morphometry study involving 15 PD patients with freezing of gait (FOG-PD) and 16 HC, no differences were found in the total volume of the cerebellum or the volumes of its lobes between the FOG-PD group and HC. No significant difference was found in the gray matter volume of the cerebellar locomotor region, fastigial nucleus, and dentate nucleus seed regions between PD patients and the HC group. However, in the subgroup comparison of the subjects, it was determined that PD patients had higher functional connectivity in these regions than the HC group. In another study using the same method, Ma et al. [25] did not report any differences in cerebellum volumes in a comparison among 37 tremor-dominant PD patient group, 17 akinetic/rigidity-dominant PD patient group, and 39 HC group. Similarly, a study by Sahin et al. [15], using stereological techniques, specifically the planimetry method, with 18 PD patients and 19 HC, showed no difference in cerebellar volumes. In contrast to these results, our study found that the cerebellum volume in the PD patient group was approximately 9% larger than that of the HC. To clarify this discrepancy, an increase in the number of subjects and further research are needed.

Lee et al. [26] have reported that no difference was observed in the comparison of ICV measured manually with MRI in a 32-member PD patient group when compared to HC and those with dementia (PDD) and without dementia (PDND) Parkinson’s disease. Laansma et al. [27], in a multicenter study involving 2357 PD patients and 1182 HC, found that the ICV value calculated using FreeSurfer was higher in the PD patient group than in HC. Despite similar results with our study, the result we found was not significant. However, the larger ICV found in the PD patient group suggests that cranial overgrowth may be a risk factor for the disease.

Maiti et al. [28] conducted unbiased cerebellum segmentation using FreeSurfer based on fMRI in 81 PD patients. Given the small individual sizes of the anatomical subparts of the vermis cerebelli, they were merged, resulting in inconsistent segmentation of the FL; therefore, this region was not considered for further analysis. Despite finding a larger volume of the FL in the HC group, the difference was not statistically significant in our study.

The human brain exhibits considerable variability in size [29]. This variability is influenced by factors such as sex and overall body size. Notably, ICV experiences a significant increase from birth to childhood, predominantly within the first 5 years of life [30]. ICV generally stabilizes between the ages of 16 and 20, and it is posited to remain constant after that [31]. Despite the occurrence of brain atrophy, ICV appears to maintain consistency. The variability introduced by factors such as age, sex, and body size presents challenges in the comparative assessment of brain atrophy or cerebral edema among individuals. To adjust for these variations, the brain-to-intracranial cavity volume ratio, called normalization, is recommended [32]. As an alternative, ICV measurements may estimate premorbid brain size in cases of neurodegenerative diseases.

When normalized values were calculated from the obtained parameters, the percentage ratio of the cerebellum’s total volume to the ICV was determined, resulting in the normalized total cerebellar volume being larger in the PD patient group compared to the HC group. According to this ratio, the total volume of the cerebellum was larger in the PD patient group. Although the normalized total volume of the FL, calculated as a percentage of the total cerebellar volume, was larger in the HC group than in the PD patient group, this difference was not statistically significant.

O’Callaghan et al. [33], in their voxel-based morphometry study of a PD patient group with an average age of 66–90, reported a loss of gray matter in the cognitive and motor regions of the cerebellum and, more importantly, identified a significant inverse correlation between cerebellar connectivity and gray matter volume. Morelli [34], in a study with 48 fallers and 63 non-fallers with PD patients, with an average age of 60–70, reported a significant decrease in the volumes of gray matter in areas of the cerebellum associated with cognitive performance but not with motor or postural performance in the PD patient group with a history of falls. In contrast to these findings, our study found that the cerebellar gray matter volume in the PD patient group was 92.37 ± 9.45 cm^3^, while it was 84.83 ± 7.76 cm^3^ in the HC group, which is a higher value in the PD patient group and this difference is significant. We believe that the discrepancy from the outcomes of studies in the literature is due to the inclusion of younger PD patients in our study.

Upon reviewing the literature, studies have yet to be encountered regarding the gray matter volume of the FL in the PD patient group. In the current study, the gray matter volume of the FL was found to be 1.14 ± 0.19 cm^3^ in the PD patient group and 1.02 ± 0.13 cm^3^ in the HC group. The value was higher in the PD patient group, and this difference is significant. This finding suggests that PD may affect the gray matter structure of the cerebellum.

When normalized values were calculated from the obtained parameters, the percentage ratio of the total gray matter volume of the cerebellum to the ICV was determined, and the normalized total gray matter volume of the cerebellum was found to be larger in the PD patient group compared to the HC group. According to this ratio, the total gray matter volume of the cerebellum was larger in PD patients. Although the normalized total gray matter volume of the FL, calculated as a percentage of the total cerebellar volume, was larger in the PD patient group than in the HC group, this difference was not statistically significant. These results suggest that while total gray matter volumes were larger in PD patients, the FL volumes did not differ between the groups. In this context, the observed increases in cerebellar volume during PD may indicate that compensation mechanisms are often activated to perform more specific functions, such as the regulation of fine motor movements and coordination, rather than balance disorders in the later stages of the disease. This supports the suggestion that the increase in cerebellum volume occurs in PD to maintain or compensate for fine motor skills rather than to support motor control and balance.

Cilia et al. [35] reported a relationship between longer disease duration and lower tremor severity. Both of these factors could be determinants of the clinical features of PD. Still, the high variability in disease duration and limited effect size make it difficult to interpret such results further [36]. Kerestes et al. [37] 2487 PD patient group reported no significant correlation between disease duration and total or regional cerebellar volume. Still, a smaller bilateral lobule VII volume was observed in the posterior lobe as the H&Y increased. Our study found no correlation between the disease duration and H&Y of PD patients and cerebellar volume data.

Aras et al. [38] analyzed the relationship between UPDRS and balance scores in patients with idiopathic PD patients and found significantly high correlation values between the functional reach test and UPDRS. Although no significant correlation was found between MDS-UPDRS3 total score and cerebellar lobule volume in another study, changes in the anterior lobe volume of PD patients were shown to match the specific motor symptoms of the disease [37]. Although total FL volume and total FL gray matter volume are not mentioned in the literature in PD patients, our study’s results show a moderate negative correlation between total FL volume, FL gray matter volume, normalized total FL gray matter volume, and UPDRS-III score. This suggests it may affect the total volume and gray matter structure of FL, a separate cerebellum lobe.

Our study has certain limitations. Considering the limited number of patients, differences in gender distributions between study groups, and insufficient clinical data, this study did not conduct subgroup analyses for PD. This characteristic can be defined as the primary limitation of our study. However, it should be remembered that previous studies have yet to find common results regarding the changes and localization of volumetric differences. In the studies in the literature, FL volume was either not analyzed or could not be analyzed because it is a small lobe. The results of the data we obtained using VolBrain show that total FL volume, FL gray matter volume, and normalized total FL gray matter volume are correlated with the UPDRS-III score. Additionally, while the elderly PD patient group has been included in the studies, our study was performed on younger patients. A significant difference was found between the groups (average age 58.91 years). To our knowledge, this study is original because it is the first to calculate the volume of the cerebellar lobule segmentation with VolBrain in PD and compare the volume of the FL between HC and PD patient groups.

There are still controversial views on whether cerebellar volume is affected in PD. Although the cerebellum is potentially related to the symptoms of PD, data on the interaction between the cerebellum and PD in the literature remain limited. To clarify the pathological changes in the cerebellum associated with PD and elucidate how cerebellar pathological and compensatory effects change as the disease progresses, further studies are needed with more subjects or more advanced imaging methods.

## Conclusion

In this study, we analyzed volumetric MRI results, comparing the cerebellum’s total volume, gray matter volume, and the volume of the FL between 24 HC and 23 PD patients. Our findings revealed that the total cerebellum volume and gray matter volume in   PD patients were larger than those in healthy individuals. These results challenge some prior reports in the literature and add a new dimension to our understanding of cerebellar involvement in PD. Moreover, our findings support the association between cognitive function in PD and specific regions in the cerebellum. Despite the variance in results from different studies, it is evident that cerebellar hyperactivity and its potential association with PD symptoms remain areas that need further exploration.
